# Growth, yield and quality of two guar (*Cyamopsis tetragonoloba* L.) ecotypes affected by sowing date and planting density in a semi-arid area

**DOI:** 10.1371/journal.pone.0257692

**Published:** 2021-09-21

**Authors:** Maryam Mahdipour-Afra, Majid AghaAlikhani, Soleiman Abbasi, Ali Mokhtassi-Bidgoli

**Affiliations:** 1 Department of Agronomy, Faculty of Agriculture, Tarbiat Modares University, Tehran, Iran; 2 Department of Food Science and Technology, Faculty of Agriculture, Tarbiat Modares University, Tehran, Iran; Anhui Agricultural University, CHINA

## Abstract

The growth period, phenology, grain yield and gum content of two different guar ecotypes were studied in response to different sowing dates and plant densities. A two-year field experiment was conducted as a split-factorial in a randomized complete block design (RCBD) with three replicates in the research field of Tarbiat Modares University during 2016 and 2017 growing season. Main plots consisted of four sowing dates (May 21, June 4, June 21 and July 5 in 2016 and May 10, May 26, June 10 and June 26 in 2017), and subplots including three plant densities (13, 20 and 40 plants m^-2^) and two ecotypes (Pakistani and Indian). Based on findings, the phenological traits, plant height, grain yield and harvest index were significantly affected by plant density. The effect of ecotypes was statistically significant (p<0.05) on all traits except harvest index in the first year. Furthermore, the seed sowings on May 21 and May 26 with 13 plants m^-2^ led to highest grain yield (3004.8 and 2826.10 kg.ha^-1^ for two consecutive years). The high gum content (33.68 and 33.78% for two consecutive years) was also recorded for Pakistani ecotype while for gravity, Indian ecotype showed higher value in both crop years. By and large, the Pakistani ecotype showed better response compared to the Indian one in both years, especially in 1^st^ and 2^nd^ sowing dates.

## Introduction

Water is considered as a unique natural resource in many areas of the world, particularly in arid and semi-arid regions including Iran [1]. It is also considered as a global limiting factor in expansion of arable lands [2]. Moreover, continuous cultivation of crops with high water demand has affected the economy of regional agriculture by increasing the cost of production. Therefore, introducing alternative crops with low water requirement and production costs could be a practical strategy towards sustainable agriculture in these regions [3].

Guar (*Cyamopsis tetragonoloba* L.) as a legume crop of warm season with the deep and well-developed root system is drought tolerant which is cultivated mainly as a rain-fed crop in arid and semi-arid areas [4]. It has a high salinity resistance [1] and requires 200 to 375 mm annual rainfall with plenty of sunshine [5]. Guar growth season ranges from 60–90 days for determinate and 120–150 days for indeterminate varieties [5]. Guar has been traditionally used as a vegetable, livestock feed and a green manure crop in agriculture [6]. It could be cultivated in poor and marginal lands and requires lower agronomic inputs. As a grain legume, it is able to symbiotic nitrogen fixation [7]. Therefore, it can improve the soil quality in a cost-effective and natural way as a consequence the yield of subsequent crops. In addition, Guar gum is a natural polysaccharide with high molecular weight, which easily hydrates in cold water to form highly viscous dispersion or even gel at low concentrations [8, 9]. The presence of glactomannan gum makes guar an important industrial cash crop with over 300 industrial applications [9].

The sowing date and plant density are the most important factors for a proper crop stand establishment in the field and potential yield [10]. Also, proper sowing date is vital for obtaining high yield due to climatic fluctuations. Guar sowing date could vary between May to August [11]. Tiwana and Tiwana [12] reported that late sowing (June 30) resulted in significantly higher plant height than early one (June 15 and 17). It is also reported that the early sowing date resulted in significantly higher grain weight compared to late sowing one [2]. Kalyani [13] revealed that among different sowing dates (first and second fortnight of July and August), the first fortnight of July was much better than others. In addition, Nandini et al. [14] reported that 10^th^ July sowing date had resulted in a higher number of pods plant^-1^ and grains pod^-1^ than any early or late sowing dates. Evaluation of 22 guar genotypes also showed that gum content was varied slightly (28.47% to 32.89%) by ecotype but not the environment Naik et al. [15]. The first fortnight of July sowing also led to significantly higher crude gum content than delayed sowings (Kalyani and Sunitha [16]. In addition, the optimum plant density with proper geometry of plant is dependent on variety, growth habits and agro-climatic conditions [17]. Siddaraju et al. [18] reported that among three plant spacing (45×15, 45×30 and 60×30 cm), the highest grain yield of guar belonged to 45×15 cm plant spacing.

Due to the relatively high tolerance of guar to drought and salinity, it can be a valuable alternative crop for the exploitation of the semi-arid area. In Iran, guar plant has been cultivated only in limited areas, in the southern parts of Sistan and Baluchestan province (Saravan, Iranshahr), as a forage crop. There is no information on guar seed production. Therefore, the aim of present research was to explore the possibility of introducing the guar into semi-arid regions like Iran and to identify suitable sowing date and plant density for augmenting the productivity and quality.

## Materials and methods

### Site description, cultural practices and experimental design

**Fig 1:**
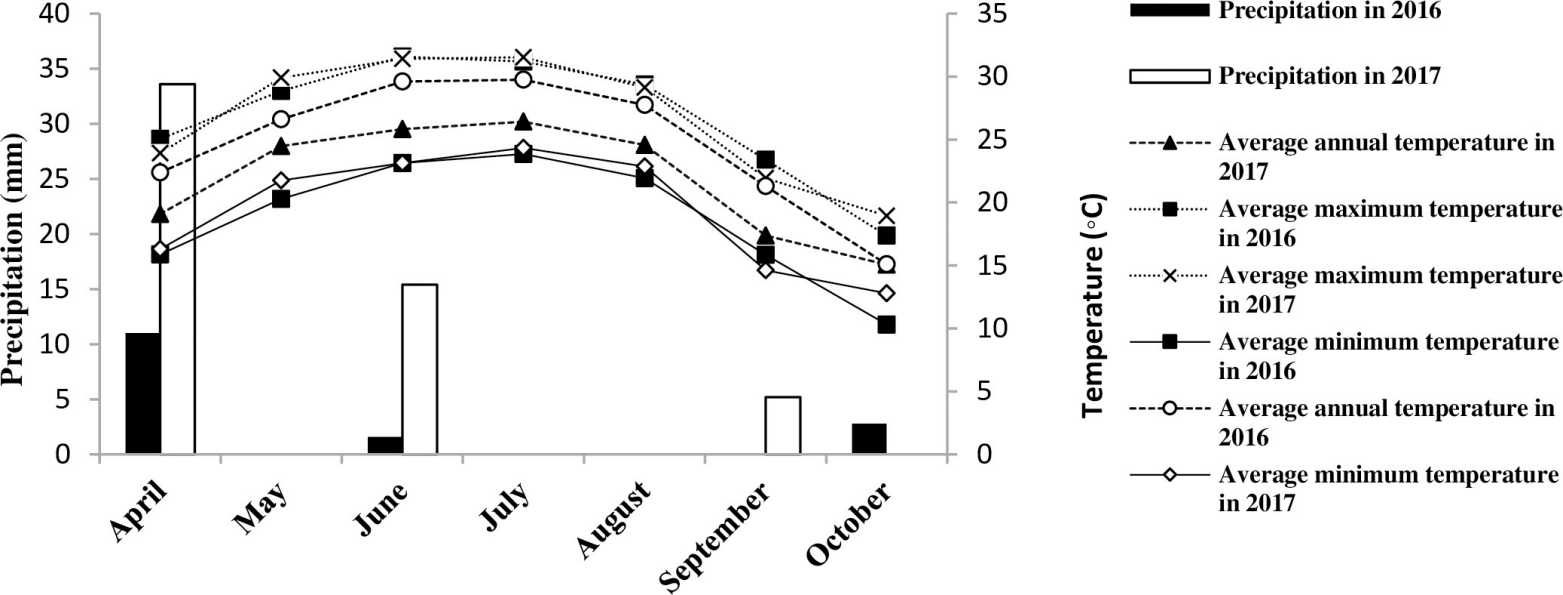
Daily temperature (°C) and precipitation (mm) records during two growing seasons (2016 and 2017).

Field experiments were conducted during two consecutive cropping seasons (2016 and 2017) in the research field of Tarbiat Modares University (39N UTM zone, Easting: 514794, Northing: 3955339), Tehran, Iran. According to the long term (30 years) rainfall (246.73 mm) and temperature (22 ˚C) records, this area is considered as semi-arid according to the De Martonne classification [1]. The weather parameters for the research field are summarized in Fig 1 (meteorological data is cited in S2 Data). The soil texture of the research field was sandy loam (Table 1).

**Table 1:** Some physico-chemical properties of the research field soil.

Parameter	Depth (cm)	
	0–30	30–60
Clay (%)	16	17
Sand (%)	60	58
Silt (%)	24	25
EC (dS.m^-1^)*	1.13	0.8
pH	7.37	7.58
T.N.V (%) **	6.5	5.5
Organic matter (%)	0.72	0.61
Total N (%)	0.13	0.11
P (ppm)	77	65
K (ppm)	412	400

*Electrical conductivity and

** Total neutralizing value.

In the present study, the effect of three factors including: sowing date, plant density and ecotype was investigated. Since sowing dates are split in time, the experiments was carried out as a split-factorial in a randomized complete block design (RCBD) with three replications. The main plots were allocated to sowing dates (May 21, June 4, June 21 and July 5 in 2016 and May 10, May 26, June 10 and June 26 in year 2017), and factorial arrangement of ecotypes (Pakistani and Indian) and plant densities (13, 20 and 40 plants m^-2^) were located in subplots. The seeds of Indian ecotype with approved name as WINNER were purchased from Nuffield Genetics PVT. LTD., Ahmedabad, Gujarat, India), also seeds of local Pakistani ecotype without approved name were obtained from Iranshahr city in Sistan and Baluchestan province, Iran, which were imported to this area from the border town of Panjgour in Baluchistan province of Pakistan for cultivation as a forage crop.

Each plot contained five rows with 4.5 m length which located 0.5 m apart of each other. Seeds planted with spaces of 15, 10 and 5 cm on each row to achieve desirable plant densities (i.e., 13, 20 and 40 plant m^-2^), respectively. Based on the soil analysis (Table 1), no phosphorus and potassium fertilizers were needed. Nitrogen fertilizer (150 kg N ha^-1^) in the form of Urea (46% N) was applied in two equal splits at the four-leaf and the beginning of stem elongation stages. The sowing was carried out in all plots in every sowing date according to the specified treatments in both years. No significant incidence of pest or disease on the crop growth was observed during the two growing seasons. Weed control was performed by hand weeding before the 3-leaf stage.

### Phenological traits

Flowering and physiological maturity were recorded on daily base observations. The number of days from seed sowing until first flower appearance at 5% (Days to flower initiation) and 50% (Days to 50% flowering) in each plot were also documented. In addition, 75% physiological maturity and pods browning were considered as "time to physiological maturity" index.

### Plant height and yield components

At physiological maturity stage, 10 plants per plot were selected randomly in order to record plant height and yield parameters (number of grains per pod, number of pods per m^2^ and 100-grain weight). The total biomass of aerial parts of plants grown in the center (1 m^2^) of each plot was harvested to determine the biomass yield. Total dry weight was recorded after oven drying at 70 ˚C to a constant weight. Grain yield were determined from oven-dried samples. The pods were then taken, threshed, cleaned with a wind blower, and weighed. The harvest index was calculated by dividing grain weight∙m^-2^ to whole plant dry weight m^-2^.

### Guar gum purification and viscosity

In first, the crude guar gum was obtained according to the protocol [8], and then was purified following a method described by Ghosh et al. [19]. In short, 5 g of crude guar gum was refluxed for 10 min under magnetic stirring in 100 mL of 80% v/v boiling ethanol. The slurry was filtered on a glass filter and washed successively with 50 mL each of ethanol, acetone and solvent ether. The precipitate was further added in 500 mL of distilled water and allowed to soak under stirring for 1 h at room temperature. The solids were collected by centrifugation for 15 min at 1500 rpm. Precipitated biopolymer was dissolved in hot water at 70°C and centrifuged at 6000 rpm for 1 h at 22°C. The supernatant was added with ethanol under external cooling and the precipitate was collected on a glass filter, washed successively with 20 mL each of ethanol and acetone. Powdered guar gum thus obtained was dried in a current of air and preserved.

A 1% (w/w) dispersion of the extracted guar gum [20] was then prepared and the viscosities were determined using a Brookfield Viscometer (model LV, Spindle No. SC4-25, 50 rpm) at 24 ˚C.

### Statistical analysis

Main and interaction effects of the experimental factors were determined employing analysis of variance (ANOVA) and using generalized linear model (GLM) in software SAS (9.1.) However, it needs to be notified that the univariate procedure was employed prior to ANOVA to ensure that the residuals were normally distributed. The existence of interactions for each measured trait meant that interpretation of the main effects was incomplete or avoiding. The interaction analysis was done by slicing in SAS. Bartlett’s test was used to test the homo-density of errors variances for all traits in two years. Due to the significant χ2 from Bartlett’s test for most traits, data were analyzed separately for each year. Means comparison of the studied traits was carried out using least significant difference (LSD) test at five percent probability level.

## Results and discussion

Analysis of variance indicated that the interaction effect of sowing date × plant density on the number of pods per square meter, grain yield and total dry matter as well as harvest index in the second crop year was significant (Table 1). The main effect of sowing date on all studied traits, except gum content and gravity in both crop years, and the main effect of plant density on all phenological traits, plant height (only in the first year), grain yield and harvest index as well as the main effect of ecotypes on all traits, except harvest index at year 1 were significant (Tables 2 and 3).

**Table 2:** Analysis of variance on morphological and phenological traits affected by sowing date, plant density and ecotype in 2016 and 2017.

		Days to first flowering		Days to 50% flowering		Days to maturity		Plant height		No. of pods per m^2^		No. of grains per pod	
S.O.V	df	2016	2017	2016	2017	2016	2017	2016	2017	2016	2017	2016	2017
B	2	ns	ns	ns	ns	ns	ns	ns	ns	ns	ns	ns	ns
S	3	**	**	**	**	**	**	**	**	**	**	**	*
S (B)	6	2.45	11.43	1.96	8.74	3.125	38.83	25.3	86.51	6477	4591	0.96	2.51
D	2	*	*	**	**	**	*	**	**	ns	ns	ns	ns
S × D	6	ns	ns	ns	ns	ns	ns	ns	ns	**	**	ns	ns
E	1	**	**	**	**	**	**	**	**	**	**	**	**
S × E	3	ns	ns	ns	ns	**	ns	**	ns	**	**	ns	ns
D × E	2	ns	ns	ns	ns	ns	ns	ns	ns	ns	.ns	ns	ns
S ×D × E	6	ns	ns	ns	ns	ns	ns	ns	ns	ns	ns	ns	ns
Error	40	2	6.55	1.44	6.76	6.9	34.31	57.65	86.67	13977	2243	0.25	2.14
C.V (%)		4.62	7.96	3.55	7.32	2.55	5.49	8.98	10.35	24.02	8.39	6.47	18.03

*,** and ns indicate significant at the p < 0.05, 0.01 levels and not significant, respectively. B: block, S: sowing date, D: density, E: ecotype.

**Table 3:** Analysis of variance on hundred grain weight, grain yield, total dry matter, harvest index, gum content and viscosity affected by sowing date, plant density and ecotype in 2016 and 2017.

		100-grain weight		Grain yield		Total dray matter		Harvest index		Gum content		Viscosity	
S.O.V	df	2016	2017	2016	2017	2106	2017	2016	2017	2016	2017	2016	2017
B	2	ns	ns	ns	ns	ns	ns	ns	ns	ns	ns	ns	ns
S	3	*	*	**	**	**	**	**	**	ns	ns	ns	ns
S (B)	6	0.11	0.09	46894	125379	828563	795364	0.002	0.001	3.64	4.14	11533	2136
P	2	ns	ns	**	**	ns	ns	*	**	ns	ns	ns	ns
S × D	6	ns	ns	**	**	**	**	ns	**	ns	ns	ns	ns
E	1	**	**	**	**	**	**	ns	*	**	**	**	**
S × E	3	ns	ns	**	**	**	**	*	ns	ns	ns	ns	ns
P × E	2	ns	ns	ns	ns	ns	ns	ns	ns	ns	ns	ns	ns
S × D× E	6	ns	ns	ns	ns	ns	ns	ns	ns	ns	ns	ns	ns
Error	40	0.14	0.08	108201	75090	701289	478945	0.003	0.001	1.09	2.97	3015	1601
C.V (%)		11.82	8.73	27.68	19.52	14.78	11.42	29.53	15.36	3.2	5.26	16.38	16.01

*, ** and ns indicate significance at p < 0.05, 0.01, and not significant, respectively. B: block, S: sowing date, D: density, E: ecotype.

Furthermore, the interaction effect of sowing date × ecotype on days to maturity (1^st^ year), plant height (1^st^ year) and the number of pods per m^2^ as well as grain yield, total dry matter (both years) and harvest index was also significant (1^st^ year). No significant interactions were found between plant density and ecotype. Furthermore, no significant three way interactions were observed in this study (Tables 2 and 3).

### Phenological parameters

The mean comparisons indicated that sowing on 21 May in 1^st^ year and 10 May in 2^nd^ year led to a flowering initiation delay in both years. Moreover, the earliest flowering initiation stage was recorded in treatments 5^th^ July in 1^st^ year and 26^th^ June in 2^nd^ year. However, no significant difference was observed between treatments 10^th^ May and 26^th^ May, in 2^nd^ year (Table 4). Fifty percent flowering stage occurred too late in treatments on 21^st^ May in 1^st^ year and 10^th^ May in 2^nd^ year and too early in treatments 5^th^ July in 1^st^ year and 26^th^ June in 2^nd^ year (Table 4).

**Table 4:** Guar phenology, and number of grains per pod traits affected by sowing date, plant density and ecotype.

	Days to first flowering		Days to 50% flowering		Days to maturity		No. of grain per pod	
Sowing date	2016	2017	2016	2017	2016	2017	2016	2017
S1	35^a^	36^a^	37^a^	40^a^	109^a^	113^a^	10^a^	9.1^a^
S2	32^b^	35^a^	35 ^b^	37^b^	106^b^	108^b^	9.2^a^	8. 9^ab^
S3	30^c^	31^b^	33^c^	35^c^	101^c^	106^b^	6.7^b^	7.6^bc^
S4	25^d^	27^c^	29^d^	30^d^	95^d^	99^c^	5.3^c^	6.8^c^
Density								
D1	31^a^	33^a^	35^a^	37^a^	105^a^	109^a^	8.9^a^	7.8^a^
D2	30^ab^	32^a^	34^b^	36^a^	103^b^	106^ab^	7.8^a^	8.4^a^
D3	30^b^	31^b^	32^c^	34^b^	101^c^	104^b^	7. 7^a^	8.1^a^
Ecotype								
E1	33^a^	33^a^	36^a^	37^a^	111^a^	115^a^	8.2^a^	8.6^a^
E2	29^b^	31^b^	32^b^	34^b^	95^b^	98^b^	7.3^b^	7.6^b^

Means within a column followed by the same letter do not significantly different from each other (P< 0.05; LSD test). S: sowing date (S1: 21^st^ May, S2: 4^th^ June, S3: 21^st^ June, S4: 5^th^ July) in 2016 and (S1: 10^th^ May, S2: 26^th^ May, S3: 10^th^ June, S4: 26^th^ June) in 2017; D: density (D1: 40 plants per m^2^, D2: 20 plants per m^2^, D3: 13 plants per m^2^) and E: ecotype (E1: Pakistani, E2: Indian).

The sowing date has been reported as a key factor to change phenologic stages of plants as found in an alteration of the number of days for flower initiation, days to 50% flowering and period of maturity [10]. In the present study, it seems that differences in plant phenological processes among the sowing date treatments might be attributed to genetic characteristics and abiotic factors, such as temperature and humidity differences. Variation in flowering and maturation time has also been observed by [21], who reported that cluster bean was a photosensitive crop for its flowers and fruits. The previous researches have revealed that days to flowering initiation and maturity of spring canola decreased due to the late seed sowing [22]. Moreover, the higher plant density delayed the flowering initiation, 50% flowering and maturity (Table 4). Between two ecotypes, Pakistani showed significantly higher values for all the phenological traits (Table 4). In other words, its flowering date and maturity stage were longer than Indian. Variation in alleles at the days to flowering initiation and maturity play an important role in providing a range of adaptation for legumes as well as affecting productivity [23].

### Agronomic traits, yield and yield components

Plant height is known as an important factor to determine competitive success for capturing the solar radiation depending upon both genetic and environmental characteristics. Significant interaction of sowing date × plant density is reported in Table 6. In case of plant height, the only significant difference was observed among treatments 13 and 40 plants per m^2^ in the latest sowing in the 1^st^ year and due to the same plant densities in the sowing date of 26^th^ May in the 2^nd^ year. In general, the shortest plants were observed in treatment 40 plants per m^2^ (Table 6). In the current study, plant height significantly decreased with delay in seed sowing and increase in plant density. It seems that, the early sown plants might have more time for absorption of water and nutrients from the soil, attaining proper vegetative growth, more efficient radiation use and development of more photosynthesis than late-planted crops. Similar finding was reported by [11], who found that May sowing date in comparison with June planting results in taller plants in Mediterranean environment of Italy. Khalil et al. [24] proposed that reduction in plant height in a delayed sowing date might be as a result of favourite months of May and June for plant growth and development. In line with the achievements of the present study, it has recently been shown that plant height decrease in a delayed sowing date can be due to the fact that the growth period of the plant is affected by the temperature rise that leads to disruption of photosynthesis, reducing the production of photosynthesis as well as the flexibility of the stem cells wall [25]. The results also showed that, Pakistani ecotype showed significantly greater plant height, in both years of the experiment and in all sowing dates. Variation in plant height among two ecotypes could be attributed to their genetic variability and phenological characteristics such as longer growth period of Pakistani ecotype. Similarly, significant variation among the guar strains for plant height was reported by [26].

Experimental data of this study indicated that the number of grains per pod and 100-grain weight traits were significantly affected by sowing date but not by plant density (Tables 2 and 3). The results revealed that the number of grains per pod significantly diminished in the delayed sowing date. However, there was no significant difference between treatments 21^st^ May and 4^th^ June in 1^st^ year and 10^th^ May and 26^th^ May in 2^nd^ year and between treatments 26^th^ May and 10^th^ June in 2^nd^ year (Table 4). In early sowing dates, the temperature was more suitable for plant growth (height, number of branch/plant and leaves) and development, especially reproductive processes, such as pollination and fertilization. These results are in agreement with the findings obtained by [27, 28]. Similarly, 100-grain weight decreased in the delayed sowing dates (Table 5). This was probably due to longer growth period in early sowing which results in the availability of better growing conditions and plants utilize nutrients, water and radiation more efficiently. There are several studies reported that grain weight of guar directly depended on the sowing dates [11, 13, 29].

**Table 5:** Grain weight, harvest index, gum content and viscosity affected by sowing date, plant density and ecotype.

	100-grain weight (g)		Harvest index		gum content(%)		Viscosity (mPa. s)	
Sowing date	2016	2017	2016	2017	2016	2017	2016	2017
S1	3.35^a^	3.35^a^	0.24^a^	0.24^b^	32.24^a^	32.82^a^	366.0^a^	275.7^a^
S2	3.34^a^	3.33^a^	0.21^b^	0.26^a^	33.26^a^	32.69^a^	302.3^a^	223.8^b^
S3	3.12^ab^	3.20^ab^	0.16^c^	0.19^c^	32.78^a^	32.92^a^	333.3^a^	248.5^ab^
S4	2.97^b^	3.0^b^	0.14^c^	0.16^d^	32.72^a^	32.51^a^	339.4^a^	251.7^ab^
Density								
D1	3.11^a^	3.17^a^	0.16^b^	0.19^b^	32.58^a^	32.35^a^	340.4^a^	258.^3a^
D2	3.25^a^	3.23^a^	0.19^a^	0.22^a^	32.91^a^	32.7^a^	328.9^a^	241.8^a^
D3	3.22^a^	3.26^a^	0.21^a^	0.23^a^	32.75^a^	33.15^a^	336.3^a^	249.7^a^
Ecotype								
E1	3.45^a^	3.32^a^	0.20^a^	0.22^a^	33.68^a^	33.78^a^	263.2^b^	196.7^b^
E2	2.94^b^	3.12^b^	0.18^a^	0.20^b^	31.82^b^	31.693^b^	407.2^a^	303.1^a^

Means within a column followed by the same letter do not significantly different from each other (P< 0.05; LSD test). S: sowing date (S1: 21^st^ May, S2: 4^th^ June, S3: 21^st^ June, S4: 5^th^ July) in 2016 and (S1: 10^st^ May, S2: 26^th^ May, S3: 10^st^ June, S4: 26^th^ June) in 2017; D: density (D1: 40 plants per m^2^, D2: 20 plants per m^2^, D3: 13 plants per m^2^) and E: ecotype (E1: Pakistani, E2: Indian).

The results of this study revealed that the number of pod per m^2^ increased with reducing plant density in the earliest sowing (21^st^ May) in the 1^st^ year and in the sowing date of 26^th^ May in the 2^nd^ year but decreased in the latest sowing (26^th^ June) due to 13 plants per m^2^ treatment in the 2^nd^ year. (Table 6). It seems that in the early sowing date, due to the better growing conditions and better plant establishment, lower density leads to less competition between plants and provides better conditions for utilization of humidity, nutrients, space and light which resulted in better growth of the plants and produces more flowers and pods per plant. However, increase in the number of pods per m^2^ was observed at high plant density in delayed planted guar crop. This trend might be attributed to late in the sowing because shortened growth period decreased vegetative and reproductive growth (plant height, number of pods per m^2^, number of grain per pod and grain weight) in the plant, but more plant populations with increasing number of plants per unit area can be compensated with the low number of pods per plant. Also, the results showed that guar ecotypes in an early sowing treatment produced more pods per area (Tables 6 and 7). This trend might be attributed to the late sowing dates result in a shortened growth period (vegetative and reproductive stages) which could decrease pod number. Similar results were found by [21], who showed that early-June sowing date significantly increased pod production than later sowing ones. In addition, Zhang et al. [30] proposed that soybean yield decrement by late sowing date was mainly due to the decrease in pod number.

**Table 6:** Some agronomic traits affected by sowing date× plant density interaction effect.

		Plant height (cm)		No. of pods per m^2^		Grain yield (kg ha^-1^)		Total dry matter (kg ha^-1^)		Harvest index	
Sowing date	Density	2016	2017	2016	2017	2016	2016	2017	2017	2016	2017
S1	D1	97^a^	94^a^	486^b^	617^a^	1117^b^	1428^b^	6173^b^	6745^a^	0.18^b^	0.20^b^
	D2	105^a^	102^a^	723^ab^	705^a^	2212^ab^	2021^a^	8276^a^	7438^a^	0.26^ab^	0.27^a^
	D3	111^a^	105^a^	831^a^	667^a^	3005^a^	1941^ab^	9862^a^	7893^a^	0.30^a^	0.24^ab^
S2	D1	88^a^	99^b^	524^a^	642^b^	1178^b^	1664^b^	6453^a^	7112^b^	0.18^a^	0.22^b^
	D2	92a	108ab	633a	721ab	1834^a^	2205^ab^	7928^a^	9434^a^	0.23^a^	0.23^b^
	D3	101^a^	115^a^	550a	802^a^	1455^ab^	2826^a^	6699^a^	8127^ab^	0.21^a^	0.34^a^
S3	D1	72^a^	73^a^	425^a^	489^a^	803^a^	981^a^	5196^a^	5217^a^	0.15^a^	0.18^a^
	D2	77^a^	85^a^	397^a^	479^a^	730^a^	1025^a^	4612^ab^	4797^a^	0.15^a^	0.20^a^
	D3	83^a^	87^a^	397^a^	438^a^	640^a^	900^a^	3732^b^	4582^a^	0.17^a^	0.19^a^
S4	D1	55^b^	64^a^	346^a^	433^a^	498 ^a^	698^a^	3668^a^	4301^a^	0.13^a^	0.15^a^
	D2	63^ab^	70^a^	318^a^	411^a^	442^a^	636^a^	3035^ab^	3616^b^	0.14^a^	0.17^a^
	D3	71^a^	76^a^	275^a^	361^b^	343^a^	520^a^	2371^b^	3423^b^	0.16^a^	0.15^a^

Means within a column related to each sowing date, with the same letters do not significantly different from each other (P<0.05; LSD test). S: sowing date (S1: 21^st^ May, S2: 4^th^ June, S3: 21^st^ June, S4: 5^th^ July) in 2016 and (S1: 10^st^ May, S2: 26^th^ May, S3: 10^st^ June, S4: 26^th^ June) in 201; D: density (D1: 40 plants per m^2^, D2: 20 plants per m^2^, D3: 13plants per m^2^).

**Table 7:** Some agronomic traits affected by sowing date× ecotype interaction effect.

		Days to maturity		Plant height (cm)		Number of pods per m^2^		Grain yield (kg ha^-1^)		Total dry matter (kg ha^-1^)		Harvest index	
Sowing dates	Ecotype	2016	2017	2016	2017	2016	2017	2016	2017	2016	2017	2016	2017
S1	E1	116 ^a^	122^a^	119^a^	107^a^	803^a^	708^a^	2706^a^	2052^a^	9152^a^	8005^a^	0.29^a^	0.26^a^
	E2	101^b^	105^b^	89^b^	94^b^	557^b^	618^b^	1516^b^	1542^b^	7055^b^	6712^b^	0.21^a^	0.23^a^
S2	E1	113^a^	116^a^	109^a^	115^a^	622^a^	814^a^	1774^a^	2756^a^	8186^a^	9546^a^	0.21^a^	0.29^a^
	E2	99^b^	100 ^b^	79^b^	100_b_	516^a^	629^b^	1204^b^	1708^b^	5867^b^	6902^b^	0.20^a^	0.24^a^
S3	E1	112^a^	115^a^	85^a^	904^a^	389^a^	466^a^	718^a^	949^a^	4427 ^a^	4816^a^	0.16^a^	0.19^a^
	E2	90^b^	96^b^	69^b^	73^b^	423^a^	471^a^	731^a^	988^a^	4600^a^	4915^a^	0.16^a^	0.19^a^
S4	E1	103 ^a^	108^a^	67^a^	78^a^	314^a^	397^a^	393^a^	598^a^	2942^a^	3732^a^	0.13^a^	0.16^a^
	E2	88^b^	90^b^	59^a^	61^a^	312 ^a^	406^a^	462.3^a^	637^a^	3108^a^	3829 ^a^	0.16^a^	0.16^a^

Means within a column related to each sowing date, with the same letters do not significantly different from each other (P<0.05; LSD test). S: sowing date (S1: 21^st^ May, S2: 4^th^ June, S3: 21^st^ June, S4: 5^th^ July) in 2016 and (S1: 10^st^ May, S2: 26^th^ May, S3: 10^st^ June, S4: 26^th^ June) in 2017; E: ecotype (E1: Pakistani, E2: Indian).

In this study, the highest grain yield in early sowing dates (21^st^ May and 4^th^ June in 1^st^ year and 10^th^ May and 26^th^ May, in 2^nd^ year) was obtained at the lowest plant density in both years, but in delayed sowing dates (21^st^ June and 5^th^ July in 1^st^ year and 10^th^ June and 26^th^ June in 2^nd^ year) there was no significant difference between plant densities. However, in the latest plant date (5^th^ July in 1^st^ year and 26^th^ of June in 2^nd^ year), the highest grain yield was obtained at the highest plant density (Table 6). Environmental conditions during the growth period affected grain yield in a significant manner [31]. The higher grain yield at the wider spacing in the early sowing and closer spacing in later sowing, attributed to improved yield attributing characters (such as grain weight, number of grains in pods and number of pods per m^2^). It seems that in the late sowing, in order to reduce grain yield loss, it is necessary to increment of plants number/unit area. Also, the results showed that, grain yield was also reduced with sowing date delay (Tables 6 and 7). Our finding are in agreement with Rajni et al. [28]. They reported low temperature, high humidity and short photoperiod could lead to low grain yield for the delayed plant. Also high temperature and long days could promote the early planted guar growth and increase the grain yield.

A study by Meftahizade et al. [29] showed that the early planting (February) of Grembite landrace lead to a higher seed yield than the other two planting dates (May and August). In another study by Meftahizade et al. [32] expressing higher seed yield for samples sown in the late planting (May 31^st^) compared to those cultivated in April 30^th^.

In addition, the total dry matter increased due to lower density of plants (20 and 13 plants per m^2^) in the earliest sowing date (21^st^ May) for the 1^st^ year and due to the same plant densities in the 2^nd^ sowing date (26^th^ May) for the 2^nd^ year. By contrast, total dry matter decreased due to lower plant density (13 plants per m^2^) in 21^st^ June, and 5^th^ July in 1^st^ year and 20 and 13 plants per m^2^ in 26^th^ June treatment in 2^nd^ year (Table 6). These results suggest that the dry matter production depends highly on the growth duration. In late sowing, unfavorable environmental conditions (low temperature and short photoperiod) shortened growth period and lead to yield reduction because of decreased accumulation of dry matter compared with the early sowing dates. Higher remobilization of resources in the early sown crop is probably linked to the higher dry matter, which represents the potential source for remobilization [33]. Furthermore, in early sowing date (21^st^ May in 1^st^ year and 10^th^ May and 26^th^ May in 2^nd^ year) in both years, the highest harvest index was obtained in lower plant density (Table 6). It might be related to the enhanced solar radiation penetration at lower plant density. It seems that the higher population intensifies inter- and intra-plant competition between vegetative and reproductive organs for assimilates. Furthermore, since reproductive buds are formed later than vegetative buds, the adverse effects of intensified competition impacts reproductive buds at the first place [10]. In this study, harvest index was also decreased with sowing date delay (Table 6). This trend might be attributed to shortening growing season with delay in sowing plant, lead to reduction in reproductive stage which decreased the number of flowers and pods per plant and finally harvest index.

Regarding the interaction of sowing date × ecotype, Pakistani ecotype showed a delayed maturity in all sowing dates during both years (Table 7). Similar results were obtained in terms of plant height. However, in the latest sowing date (5^th^ July in 1^st^ year and 26^th^ June in 2^nd^ year) no significant difference was detected between Pakistani and Indian ecotypes (Table 7). In first year, and under treatment sowing 21^st^ May, Pakistani ecotype produced more pod per m^2^ in comparison to the Indian one. Similar results were achieved in the second year under treatments sowing 10^th^ May and 26^th^ May (Table 7). In both years, and in early sowing dates (21^st^ May and 4^th^ June in 1^st^ year and 10^th^ May and 26^th^ May in 2^nd^ year), Pakistani ecotype produced higher grain yield and total dry matter in comparison to the Indian one (Table 7). In general, among the two ecotypes of guar in early sowing dates, Pakistani one showed significantly higher values for all traits.

### Gum content and viscosity

Guar grain contains 25–35% of galactomannans or so-called guar gum with commercial importance. According to the findings of the present study (Table 5), gum content and gravity were not affected by plant density and sowing date. However, the minimum gravity was observed in treatment sowing 26^th^ May in 2^nd^ year (Table 5). A significantly higher gum content (%) in both years was recorded with Pakistani ecotype while for gravity trait, Indian ecotype showed higher values in both years. Similarly, a significant variation among different guar landraces traits such as seed weight, seed yield, gum (%) and viscosity were reported by [32].

In general, Pakistani ecotype showed better response as compared with Indian ecotype in both years of the experiment, especially in early sowing treatments (21^st^ May and 4^th^ June in 1^st^ year and 10^th^ May and 26^th^ May in 2^nd^ year). In other words, Pakistani ecotype due to its better genetic characteristics (longer flowering date and maturity stage, higher height and leaf area index, having sub-branches) increases the amount of photosynthesis and produces more grains per pod, heavier grains and higher gum content (Tables 4 and 5). Many studies have also been reported significant genotypic variation for guar grain yield [11, 13]. Nandini et al. [14] found that variation among guar genotypes in harvest index likely occurred owing to the higher partitioning and translocation of photosynthesis from source to sink because of higher vegetative growth and higher interception and utilization of solar radiations.

## Conclusions

In this study, the possibility of introducing the guar as a grain legume crop with various industrial and pharmaceutical-cosmetic applications in the semi-arid climate of Tehran, Iran was investigated. Our findings provided new information about the effect of sowing date and plant density on growth and quantitative and qualitative yield of guar in these areas. The effect of plant density was significant in most of the studied traits, including phenological ones and plant height. Also, differences in grain yield, total dry matter and the harvest index were significantly affected by sowing date × plant density interaction. This shows that achieving high grain yield of guar depends on choosing the appropriate density and of course depends on the sowing date. Accordingly, early planting (21–26 May) was suitable sowing date for guar cultivation in such a climate. Ecotypes× sowing date interaction was statistically significant for guar grain yield, biomass, harvest index, plant height and days to maturity. Altogether, It can be concluded that Pakistani ecotype performed slightly better than Indian one in all traits except in viscosity. Based on the findings of this study, in order to maximize grain yield of Pakistani ecotype, seed sowing on 21–26 May with plant density of 13 plants per m-2 could be recommended. Generally, current results indicated that guar ecotype which evaluated for the first time in this region performed well and have provided a basic information that there is a hope for guar as an alternative crop in semi-arid region of Iran.

## Supporting information

S1 Data (XLSX)

S2 Data (XLSX)
